# Chronic SIV infection induces differentiation and accumulation of cytotoxic CD16+ NK cells in lymph nodes followed by transmigration to the mucosae

**DOI:** 10.1186/1742-4690-9-S2-P179

**Published:** 2012-09-13

**Authors:** H Li, T Evans, J Gillis, R Reeves

**Affiliations:** 1 NEPRC, Harvard Medical School, Southborough, MA, USA

## Background

Natural killer (NK) cells inhibit lentiviral replication both directly and indirectly, but substantial evidence also indicates HIV/SIV can induce NK cell dysfunction. NK cells can be subdivided based on expression of CD56 and CD16. In blood, cytotoxic CD16+ NK cells are the dominant subpopulation, while cytokine-secreting CD56+ and double-negative (DN) NK cells are the primary NK cells found in lymph nodes (LN). Furthermore, CD56+ and DN NK are thought to be precursor populations, whereas CD16+ NK cells are terminally differentiated. The effects of HIV/SIV infection on NK cell distribution, trafficking, and development are unclear.

## Methods

Macaque NK cells were isolated from blood, LN, and mucosal tissues of naive and chronically SIV-infected animals and then analyzed phenotypically by surface and intracellular flow cytometry, evaluated functionally by ICS and in a direct killing assay against MHC-devoid 721.221 cells. In situ analyses were performed by immunohistochemistry.

## Results

In peripheral blood of chronically infected animals, we found a specific expansion of CD16+ NK cells, coupled with high frequencies of cytotoxic perforin+ CD16+ NK cells in LN, where they are normally absent. Interestingly, classic LN-trafficking molecules, CD62L and CCR7, were downregulated to undetectable levels on blood and LN CD16+ NK cells, suggesting they did not migrate from extralymphoid tissues. Furthermore, the putative NK cell precursors, CD56+ and DN NK cells, exhibited increased proliferation and activation, providing a potential source of differentiated CD16+ NK cells. CD16+ NK cells in LN also upregulated the mucosa-trafficking marker, α4β7, correlating with increased frequencies of cytotoxic CD16+ NK cells in colorectal and jejunum tissues of infected animals.

## Conclusion

Our data suggest a novel mechanism whereby lentivirus infection induces differentiation of cytotoxic CD16+ NK cells, which are normally absent in LN, to differentiate in situ and then transmigrate to the gut mucosa, the primary site of virus replication.

